# Compound Fu brick tea modifies the intestinal microbiome composition in high‐fat diet‐induced obesity mice

**DOI:** 10.1002/fsn3.1850

**Published:** 2020-08-31

**Authors:** Caibi Zhou, Xiaolu Zhou, Zhirui Wen, Liming Liu, Zaibo Yang, Lu Yang, Ping Li, Xiying Guo, Xin Mei

**Affiliations:** ^1^ College of Biological Science and Agriculture Qiannan Normal University for Nationalities Duyun China; ^2^ College of Horticulture and Landscape Architecture Hunan Agricultural University Changsha China

**Keywords:** compound Fu brick tea, high‐fat diet, high‐throughput sequencing, intestinal microbiota

## Abstract

Compound Fu Brick Tea (CFBT), which is from Duyun city in China, is a traditional Chinese dark tea, Fu Brick Tea, mixed with six herbal medicine. It is consumed by local people for reducing weight, but the mechanism is not clear. The disorder of intestinal microbiome caused by long‐term high‐fat diet (HFD) is one of the inducements of obesity and related metabolic syndrome. In this study, mice were fed with HFD to establish a high‐fat model. Fifty mice were randomly divided into six groups: normal control (CK), HFD model control (NK), positive control with medicine (YK), CFBT groups with low, middle, and high dose (FL, FM, FH). The V3‐V4 DNA region of fecal microbiome from mouse intestine was sequenced. The results showed that the diversity of intestinal microflora was highest in CK and lowest in NK. Compared with CK, the dominant bacterium *Firmicutes* was increased and *Bacteroidetes* decreased at phylum level in NK. Compared with NK, the abundance of microbiome in CFBT groups was significantly higher and the composition was changed: *Muribaculaceae*, *Bacteroidaceae,* and *Prevotellaceae* increased and *Lachnospiraceae* decreased in CFBT groups at family level, while at the genus level, *Bacteroides* increased and *Lactobacillus* decreased. These results conclude that CFBT can increase the abundance of intestinal microbiome in mice, promote the growth of beneficial bacteria and reduce the number of pathogenic bacteria, and restore the imbalance of intestinal microbiome caused by poor diet.

## Graphical abstract

Compound Fu Brick Tea can increase the abundance of intestinal microbiome in mice, promote the growth of beneficial bacteria and reduce the number of pathogenic bacteria, and restore the imbalance of intestinal microbiome caused by high‐fat diet.

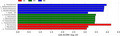

## 1 INTRODUCTION

Intestinal microbiome is the highest and most complex symbiotic microbial ecosystem in human body (host) (Peck et al., 2019), which plays an important role in the stability of intestinal environment (Nie et al., 2019). Disorder of intestinal microbiome is the cause or contributing factor of many kinds of body system diseases, including digestive system, immune system, cardiovascular system, respiratory system, and even nervous system. Genetic and environmental factors of the host will lead to differences in the composition and function of intestinal microbiome. In fact, gut provides a nutritious habitat for symbiotic bacteria, while gut microbiome makes up for the limited ability of the host to decompose the polysaccharide in the food and enhances the host's defense ability against invasive pathogens through the competition mechanism. Intestinal microbiome also affects the physiological process of the body by regulating the expression of host genes (Ai, Pan, Li, Wu, & Xia, 2019). The typical functions of intestinal bacteria are to decompose food residues, regulate the immune system, synthesize vitamins and amino acids, and metabolize drugs (Shivaji, 2019). By regulating the intestinal microbiome in the human body, it can promote digestion, improve appetite, enhance the immune capacity of the intestinal tract, increase insulin resistance (Cani, 2019), resist pathogenic bacteria, and then reduce fatty liver and hyperlipidemia.

As early as the late 1940s, obesity has been defined as a disease by World Health Organization (WHO), and has been added to the International Classification of Disease (ICD). In recent years, however, people have begun to realize the seriousness of the problem. More and more evidences show that the change of intestinal microbiome composition is related to obesity and relative metabolic disorders (Ridaura et al., 2013). Intestinal microbiome plays an important role of metabolism in host health, such as maturation of host immune system and protection of pathogens. Intestinal microorganisms can produce short‐chain fatty acid acetate, propionate, and butyrate, which are used as carbon sources by the host (Heinken & Thiele, 2015). Diet is one of the main factors to shape the structure of intestinal microbiome (Ibrahim & Anishetty, 2012; Xiao et al., 2014). It has been proved that the weight‐reducing mechanism of natural products is closely related to the regulation of intestinal microbiome. The regulation of intestinal microbiome through plant diet intervention is considered as an effective measure to treat obesity. At present, many foods and drugs have been found to regulate intestinal microbiome, but up to now, the targeted regulation of intestinal microbiome by drugs is still in the research stage. In this study, compound Fu brick tea (CFBT) was used to change the structure of intestinal microbiome, to provide new options for the prevention and treatment of obesity and related metabolic syndrome, and to provide new methods and ideas for individualized medical treatment of obesity.

Tea is rich in polyphenols, amino acids, polysaccharides and other substances, containing antioxidant, anti‐tumor, anti‐radiation, hypoglycemic, hypolipidemic, regulation of intestinal microbiome, and other health care effects. In recent years, it has been found that tea polyphenols can maintain the health of the intestine and stomach mainly by regulating the composition of intestinal microorganisms, which can promote the growth and proliferation of intestinal beneficial bacteria, including *Lactobacillus*. Meanwhile, it play a role of prebiotics in inhibiting the growth of certain pathogens, such as *Salmonella* and *Helicobacter pylori* (van Duynhoven et al., 2011). Secondly, tea can also regulate intestinal microbial structure, indirectly participate in host energy absorption, food metabolism, and even gene expression and immune response, and finally play a role in regulating obesity and related diseases (Backhed et al., 2004). In addition, tea polyphenols and other bioactive substances in green tea can reduce fasting blood glucose level and mesenteric fat, and increase serum insulin level, which may achieve the goal of lowering blood lipid by preventing β cell damage and changing the bacterial community structure in the intestine (Chen et al., 2019). Fu brick tea (FBT) is a kind of post‐fermented dark tea. It has a unique flavor and is rich in tea polyphenols, flavonoids, amino acids, and other active substances. It has the health functions of helping digestion, regulating intestines and stomach, anti‐oxidation, protecting liver, lowering blood sugar, and reducing fat and weight (Du, Wang, & Yang, 2019; Fu et al., 2011). In order to improve the flavor and function of FBT, we added 6 plants selected from *Chinese Pharmacopoeia* to make a compound tea. CFBT was prepared with FBT as the main raw material, supplemented with hawthorn, mint, chamomile, tangerine peel, fennel, and dandelion root. These Chinese herbal medicines also have health functions of regulating intestines and stomach, protecting liver and gallbladder, lowering blood fat, blood pressure and cholesterol (Amsterdam, Li, Xie, & Mao, 2019; Kapp et al., 2020; Lis et al., 2019; Lone, Parray, & Yun, 2018; Özbek et al., 2003; Zhang et al., 2002). In our previous study, the formula has been optimized by orthogonal experiments and proved that the mixed formula was as safe as its single component through the toxicological experiment in mice (Zhou et al., 2020).

At present, there are many studies on the functional components of tea, but there are few reports on the regulation of intestinal microbiome diversity by compound tea. In this study, a hyperlipidemia model was established by feeding SPF mice with high‐fat diet (HFD). The CFBT extract was infused into the stomach. The influence of CFBT on the intestinal microbiome diversity of HFD‐induced mice was studied by using Illumina high‐throughput sequencing technology. The results will provide new insights in the mechanism of CFBT for the regulation of intestinal microbiome and its effect on the prevention and control of intestinal microbiome‐related diseases.

## 2 MATERIALS AND METHODS

### 2.1 Materials

CFBT includes Fu brick tea, hawthorn, chamomile, dandelion root, mint, tangerine peel, and fennel, which are all provided by Changsha Dekang Biotechnology Co., Ltd., China. Its formulation has been reported in our previous paper (Zhou et al., 2020). The SPF male Kunming mice were provided by Hunan Shrek Jingda experimental animal Co., Ltd. (China) with license No.: scxk (Hunan) 2016–0002. The high‐fat diet was made of the following ingredients: cholesterol 1.5%, lard 10.0%, yolk powder 5.0%, bile salt 0.5%, and conventional feed 83.0%. Xuezhikang capsule, a drug for the positive control, is purchased from Beijing Beida Weixin Biotechnology Co., Ltd., China.

### 2.2 Tea Sample Preparation

CFBT was extracted in boiling water twice with mass ratio 1:10 and 1:8, respectively for 30 min and 20 min, respectively. Tea infusion was combined and filtered with two layers of industrial gauze and then with vacuum filtration. The filtrate is concentrated to a certain concentration with a rotary evaporator. After being frozen at −80°C for 12 hr, it was freeze‐dried for 24 hr, and the dry powder was collected, sealed and stored at ‐ 80°C for standby.

### 2.3 Animal Experiment

After 7 days of adaptive feeding, 60 five‐week‐old SPF male KM mice were randomly divided into 6 groups with 10 in each group: normal group (CK), model group (NK), positive group (YK), compound Fu brick tea low‐dose group (FL), medium‐dose group (FM), and high‐dose group (FH). CK group was fed with basic diet; other groups were fed with high‐fat diet. CK and YK were infused intragastrically with purified water; YK group was established by intragastric infusion of positive drug Xuezhikang (90.0 mg·kg^‐1^·D^‐1^); FL, FM, and FH group were administered intragastrically by different doses of CFBT extract (247.5, 495.0, and 990.0 mg·kg^‐1^·D^‐1^, respectively). The daily dosage of Xuezhikang Capsule for adults is 10 mg/kg, and the conversion coefficient between mice and human is 9.1, that is, 91 mg/kg in mice. The mice were infused with 0.1 ml/10 g. The extraction rate of CFBT was about 33%. The recommended amount of dry tea for adults is 9 g/day. An adult's weight was assumed to be 60 kg (equivalent to 150 mg tea per kg), and the low, medium, and high doses for mice were set to be 5, 10, and 20 times of the recommended amount, respectively, that were, 150 × 5 × 33% = 247.5 mg/kg, 150 × 10 × 33% = 495 mg/kg, and 150 × 20 × 33% = 990 mg/kg. During the experiment, the feeding temperature of each group was 20 ~ 26°C, and the relative humidity was 50%~60%. The weight, activity, and physiological status of mice were observed and recorded every week. After the modeling, the feces of mice from each group were collected aseptically, put into 1.5 ml sterilized centrifuge tube, and then frozen at −80°C for further detection. All animal experiments were conducted in the animal experiment center of Hunan Agricultural University (Hunan, China).

### 2.4 Genomic DNA Extraction and PCR Amplification of Fecal Microbiome

One hundred milligram of fecal sample was centrifuged, and 1.4 ml fecal sample lysis buffer (ASL) was added to the sample, mixed evenly. Total DNA of microorganism in the sample was extracted with GENEWIZ fecal DNA extraction kit according to its instructions. Using 30–50 ng DNA as templates, V3 and V4 regions were amplified with the upstream primer CCTACGGRRBGCASCAGKVRVGAAT and the downstream primer GGACTACNVGGGTWTCTAATCC. In addition, connectors with index were added by PCR to the ends of the PCR products of 16S rDNA for the next generation sequencing. The raw data have been deposited in the NCBI Sequence Read Archive database with the BioProject accession number PRJNA636968.

### 2.5 Sequencing and Bioinformatics Analysis

The quality of the library was detected by using the Agilent 2,100 biological analyzer (Agilent Technologies, Palo Alto, CA, USA), and the concentration of the library was detected by Qubit2.0 Fluorometer (Invitrogen, Carlsbad, CA, USA). After the DNA library was mixed, the sequencing of PE250/300 pair ends was carried out according to the operation manual of Illumina MiSeq instrument (Illumina, San Diego, CA, USA), and the sequence information was read by MiSeq Control Software (MCS) and analyzed by the GENEWIZ Bioinformation Platform (https://www.genewiz.com.cn/).

## 3 RESULTS

### 3.1 Sample Sequence and OTU Cluster Analysis

A total of 1,214,686 effective 16S rRNA gene sequences (raw reads) were obtained from 18 samples in 6 groups through high‐throughput sequencing based on Illumina MiSeq platform. After screening, filtering, optimization, removal of chimeras, and quality control of the spliced sequences, a total of 984,972 high‐quality sequences and 3,668 OTUs, with an average of 67,482 sequences and 203 OTUs for each sample were obtained. Among them (Table 1), CK group (CK1_FB ~ CK3_FB) had 126,533 sequences and 686 OTUs, FH group (FH1_FB ~ FH3_FB) 177,180 sequences and 526 OTUs, FL group (FL1_FB ~ FL3_FB) 180,315 sequences and 625 OTUs, FM group (FM1_FB ~ FM3_FB) 185,725 sequences and 614 OTUs, NK group (NK1_FB ~ NK3_FB) 149,518 sequences and 585 OTUs, YK group (YK1_FB ~ YK3_FB) 165,701 sequences and 632 OTUs.

**Table 1:** Statistics of sample data and OTU numbers

Sample	Raw reads	Final reads	OTUs
CK1_FB	41,105	31,931	212
CK2_FB	53,507	43,568	239
CK3_FB	66,881	51,034	235
FH1_FB	61,467	49,257	170
FH2_FB	68,151	62,058	154
FH3_FB	80,393	65,865	202
FL1_FB	69,768	49,658	224
FL2_FB	73,615	57,122	213
FL3_FB	84,132	73,535	188
FM1_FB	72,067	56,414	217
FM2_FB	89,167	69,703	193
FM3_FB	74,544	59,608	204
NK1_FB	61,254	49,908	177
NK2_FB	52,906	44,321	198
NK3_FB	60,807	55,289	210
YK1_FB	50,510	42,059	194
YK2_FB	76,126	62,163	216
YK3_FB	78,286	61,479	222

**Figure 1:**
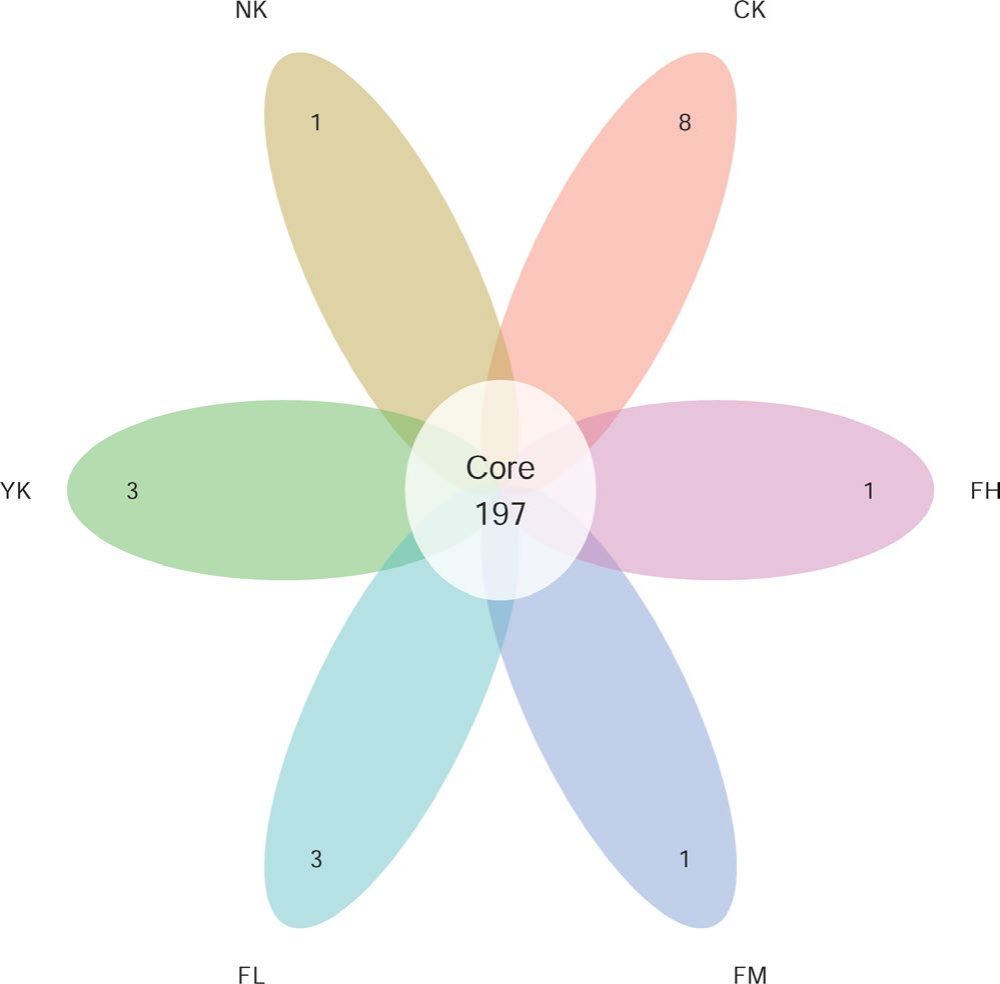
Venn plot of OTUs. Each petal represents a group, the numbers on the petals represent the numbers of species unique to the group, and the white circle in the middle represents the number of species shared by all groups

The number of unique and shared OTUs was analyzed by Venn diagram. The overlap and similarity of microbial composition have been compared in Figure 1. There were 197 species in 6 groups, including 8 specific species in CK, 1 specific species in FH, 1 specific species in FM, 3 specific species in FL, 3 specific species in YK, and 1 specific species in NK. CK group had the most abundant species, which was higher than other groups.

**Figure 2:**
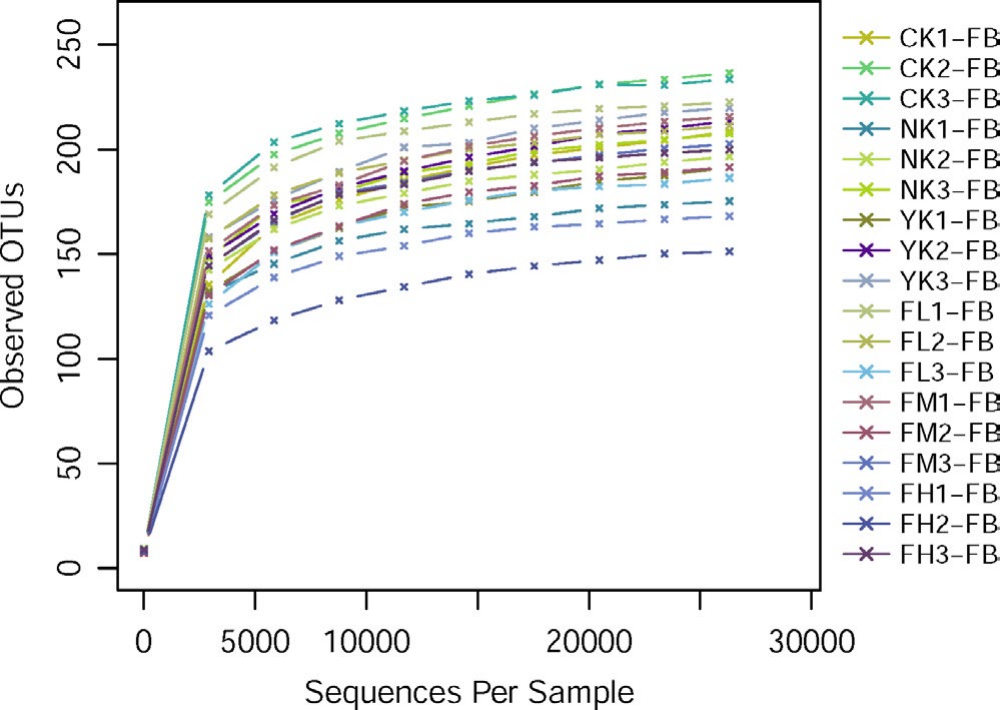
Rarefaction curves of OTUs. The abscissa is the number of effective sequences per sample and the ordinate is the number of observed OTUs. The curves reflect the increasing speed and trend of new species observed with the increase of sequencing depth in the process of sequencing samples

In order to judge whether the sequencing data were sufficient, a rarefaction curve was drawn on the basis of OTUs with 97% similarity. As shown in Figure 2, the sample size used in this study was 18. With the deepening of sequencing data, the numbers of OTUs increased dramatically. But the increasing trends of OTUs among groups were similar, and gradually tended to be horizontal, which indicated that although the amount of sequencing data continued to increase, the numbers of OTUs obtained were limited. So, the amount of data was enough to cover all species in our samples and was sufficient for further analysis.

### 3.2 Alpha Diversity Analysis of Microbiome

**Figure 3:**
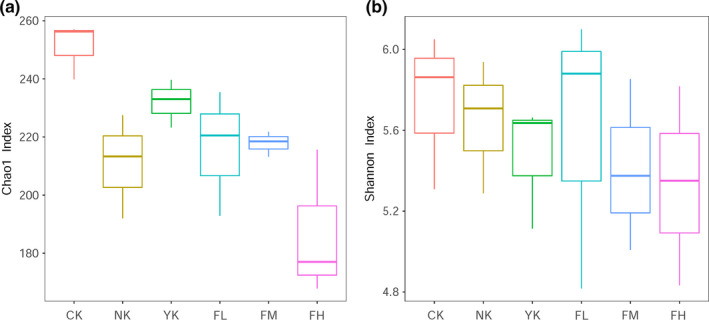
Alpha diversity illustrates the difference between samples. (a) Chao1 index; (b) Shannon index. The box chart represents the minimum value, the lower quartile, the median, the upper quartile, and the maximum value from the bottom to the top

The diversity of fecal microbiome was measured by analyzing the alpha diversity index, which were Chao1 index and Shannon index (Figure 3). Compared with CK group, the number of OTUs in other five groups decreased, especially in FH group. After the treatment with different doses of CFBT, the richness of fecal microbiome decreased and was less with the increase of CFBT dosage, indicating that CFBT can significantly change the fecal microbiome richness of mice.

### 3.3 Principal Coordinate Analysis of Microbiome

**Figure 4:**
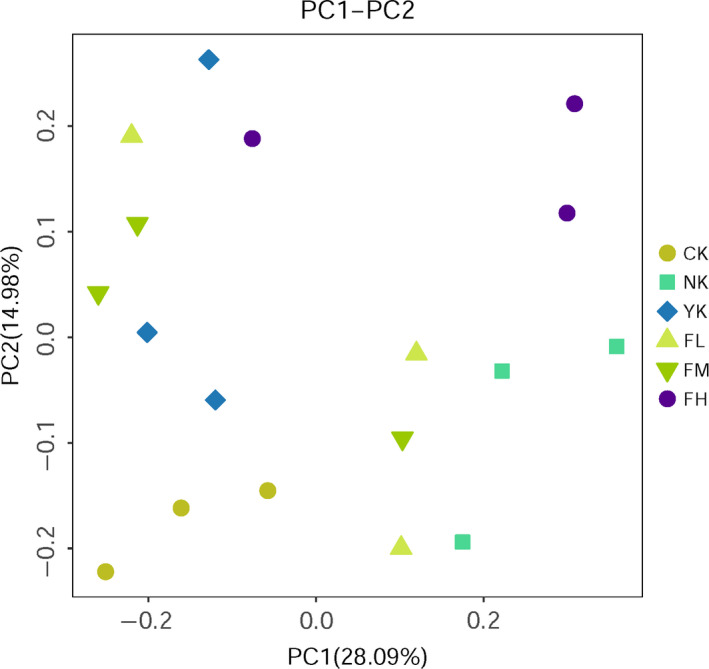
PCoA diagram of samples at the genus level of intestinal microbiota

Through principal coordinate analysis (PCoA), we can see that the samples of CK group and other five groups are distinguished (Figure 4), which indicated that the species composition of CK group was quite different from other five groups. For the CK, NK, and FH group, the mice samples in each group gathered together to form an independent area, and the difference between the groups was greater than that in the groups. The YK group was closer to the CK group than the NK group, while the FH group was closer to the NK group. For the YK, FL, and FM groups, the samples in each group could not completely gather to form an independent area due to the sampling difference. The distance between CK group and CFBT‐treated group was similar with the distance between CK and FM. All the CFBT‐treated groups were significantly different from the NK group, which can be explained that CFBT treatment was the main reason for the difference.

### 3.4 Cluster Analysis of Fecal Microbial by UPGMA Tree

**Figure 5:**
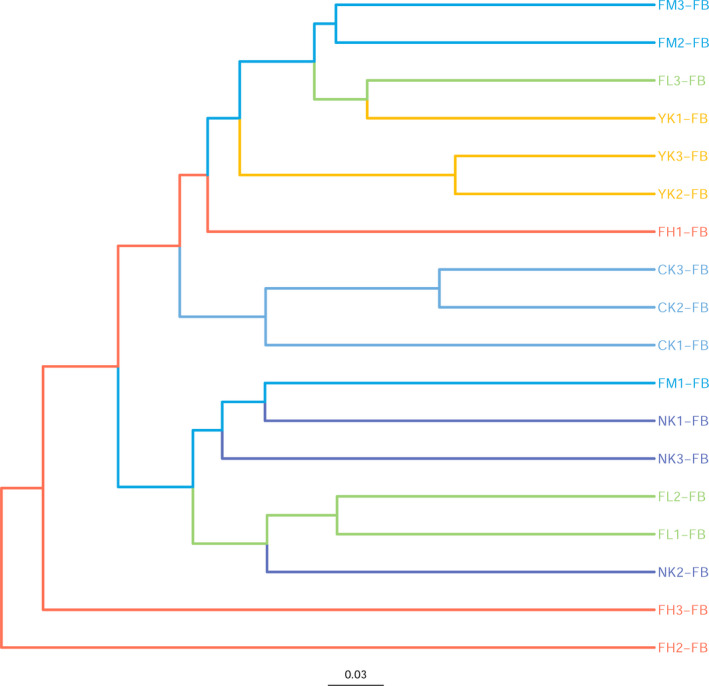
UPGMA‐tree diagram of samples

Through UPGMA‐tree cluster analysis (Figure 5), it was clear that the mice in CK group and CFBT treatment groups were distributed in totally different branches, which indicated that there were significant differences between them. The samples from CK, NK, and YK groups also belonged to different cluster branches, which indicated that they were significantly different from each other, too. Theoretically, the samples in each group should belong to the same branch, but because of the sampling difference, only YK and CK samples had good biological repeatability, and there was a deviation sample in each other group.

### 3.5 Structure and Abundance Changes of Microbiome

#### 3.5.1 Changes of Microbiome Structure and Abundance at Phylum Level

**Figure 6:**
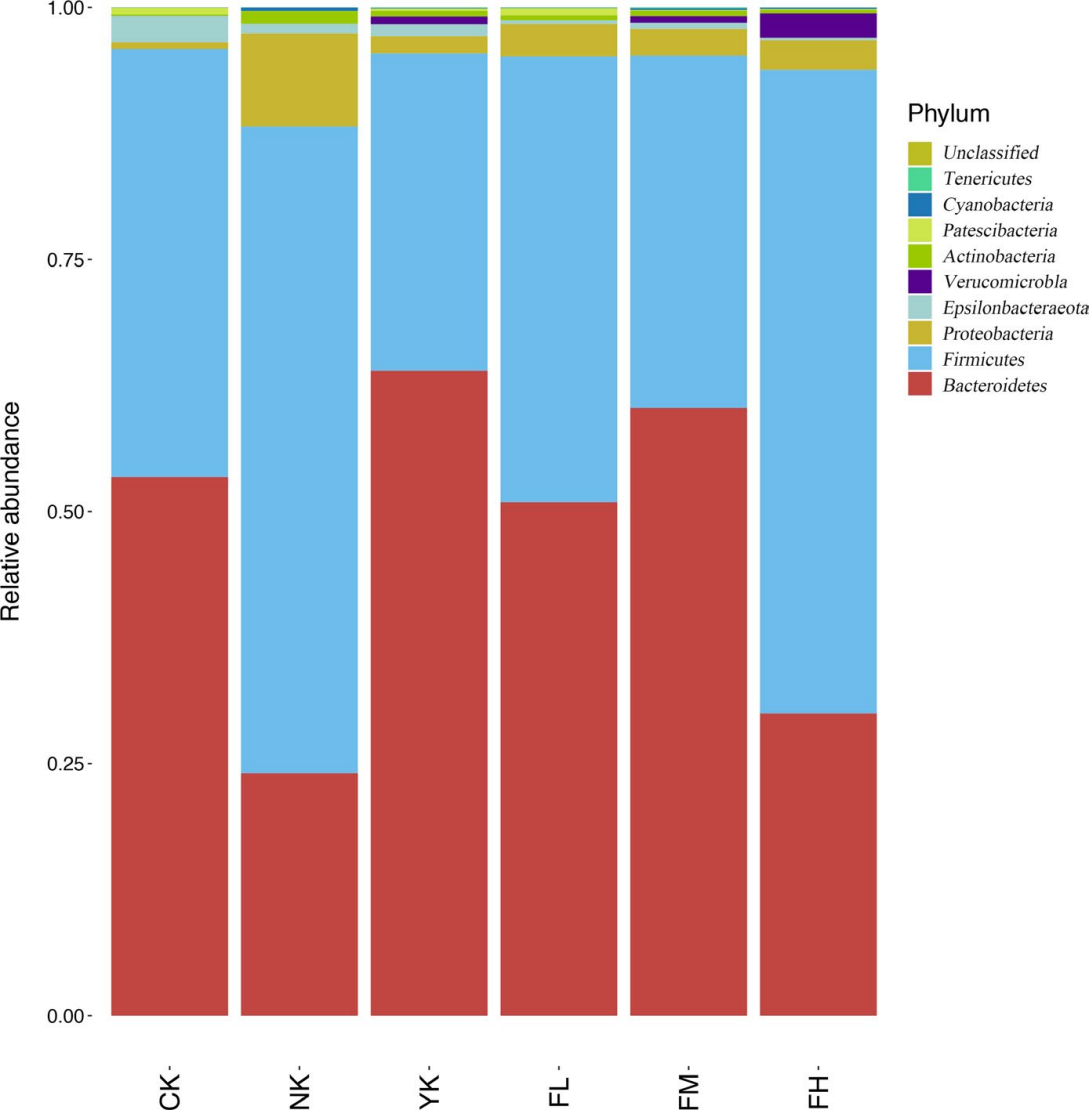
Changes of microbiome structure and abundance at phylum level

From the histogram of community structure composition at the phylum level (Figure 6 and Table 2), it was found that *Firmicutes* and *Bacteroidetes* were the dominant phylum of fecal microbiome in all mice. Other important phylums were also detected, such as *Actinobacteria*, *Proteobacteria*, *Tenericutes,* and *Verrucomicrobia*. The dominant phylums in CK group were *Bacteroidetes* (53.44%), *Firmicutes* (42.44%), and *Epsilonbacteraeota* (2.62%). The dominant phylums in NK group were *Bacteroidetes* (24.04%), *Firmicutes* (64.14%), and *Proteobacteria* (9.22%). The dominant phylums in YK group were *Bacteroidetes* (63.98%), *Firmicutes* (31.46%), and *Proteobacteria* (1.74%). The dominant phylums in FL group were *Bacteroidetes* (50.95%), *Firmicutes* (44.18%), and *Proteobacteria* (3.24%). The dominant phylums in FM group were *Bacteroidetes* (60.29%), *Firmicutes* (34.91%), and *Proteobacteria* (2.65%). The dominant phylums in FH group were *Bacteroidetes* (29.99%), *Firmicutes* (63.84%), and *Proteobacteria* (2.95%). Compared with the phylums in the CK group, *Firmicutes* and *Bacteroidetes* were still the dominant phylums in CFBT‐treated groups, but the content of *Epsilonbacteraeota* decreased and the content of *Proteobacteria* increased, which indicated that CFBT treatment could affect the content of *Epsilonbacteraeota* and *Proteobacteria* in mouse's intestine. For different concentrations of CFBT treatment, high concentration seriously reduced the content of *Bacteroidetes*, middle concentration seriously reduced the content of *Firmicutes*, which meant that the response of *Firmicutes* and *Bacteroidetes* to different concentrations of CFBT was not the same. Compared with CK group, NK and YK groups also contained the main phylums of *Firmicutes* and *Bacteroidetes*, but the content of *Epsilonbacteraeota* decreased and the content of *Proteobacteria* increased, indicating that the treatment could affect the content of *Epsilonbacteraeota* and *Proteobacteria*.

**Table 2:** Distribution of microbiome at phylum level

Taxon	CK	NK	YK	FL	FM	FH
*Bacteroidetes*	53.44	24.04	63.98	50.95	60.29	29.99
*Firmicutes*	42.44	64.14	31.46	44.18	34.91	63.84
*Proteobacteria*	0.69	9.22	1.74	3.24	2.65	2.95
*Epsilonbacteraeota*	2.62	0.98	1.17	0.37	0.62	0.24
*Verrucomicrobia*	0	0.00	0.78	0.00	0.67	2.43
*Actinobacteria*	0.14	1.24	0.53	0.50	0.51	0.31
*Patescibacteria*	0.64	0.01	0.23	0.68	0.06	0.08
*Cyanobacteria*	0.02	0.35	0.06	0.04	0.18	0.14
*Tenericutes*	0.01	0.01	0.05	0.04	0.09	0.02
Unclassified	0	0	0.00	0.00	0.00	0.00

#### 3.5.2 Changes of Microbiome Structure and Abundance at Family Level

**Figure 7:**
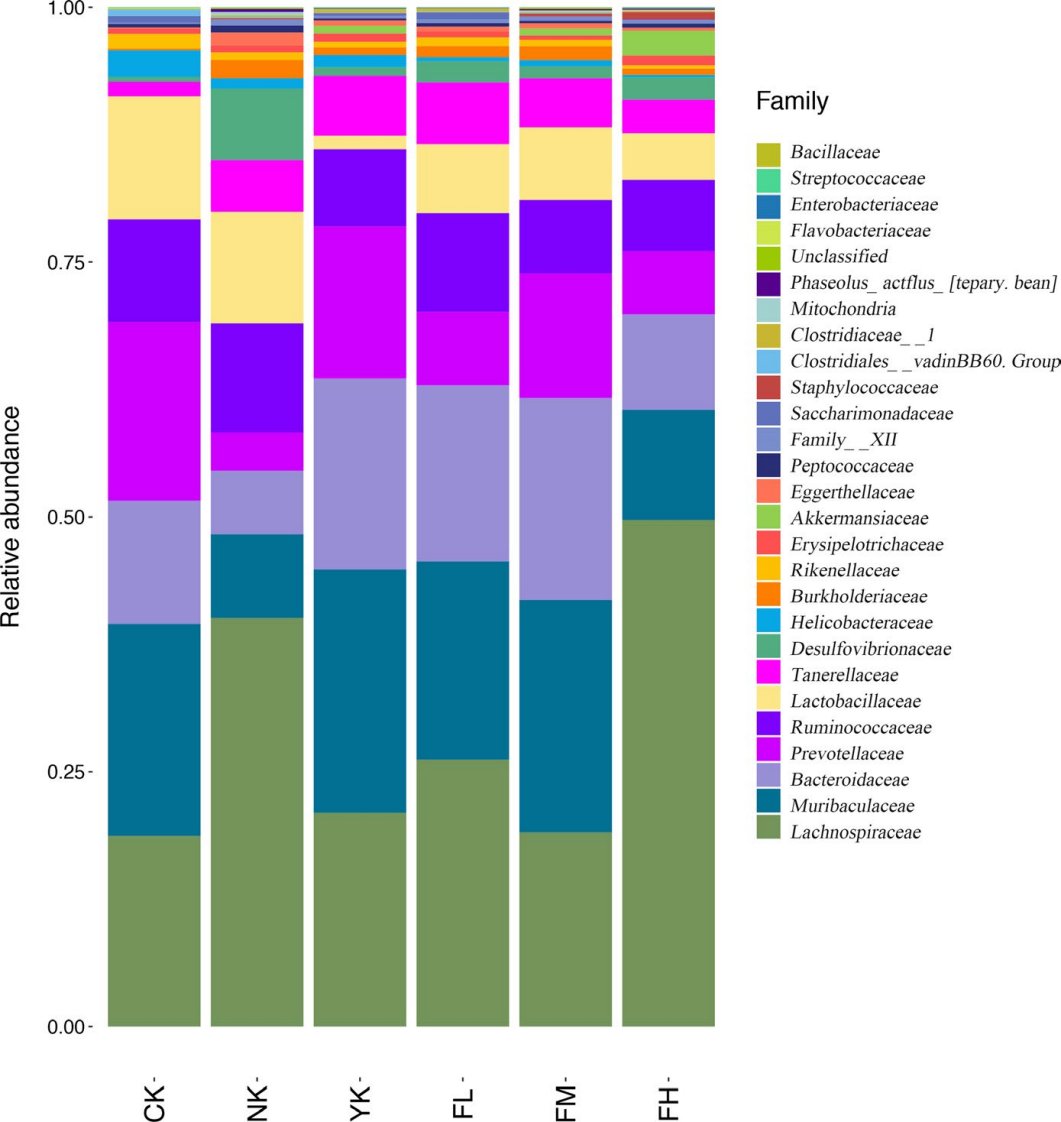
Changes of microbiome structure and abundance at family level

From the histogram of community structure composition at the family level (Figure 7 and Table 3), *Lachnospiraceae* and *Muribaculaceae* were the dominant families of all mouse fecal microbiome. The main families in CK group were *Muribaculaceae* (20.80%), *Lachnospiraceae* (18.69%), *Prevotellaceae* (17.53%), and *Bacteroidaceae* (12.11%). The main families in NK group were *Lachnospiraceae* (40.09%), *Lactobacillaceae* (10.94%), *Ruminococcaceae* (10.70%), and *Muribaculaceae* (8.20%). The main families in YK group were *Muribaculaceae* (23.89%), *Lachnospiraceae* (20.96%), *Bacteroidaceae* (18.74%), and *Prevotellaceae* (14.92%). The main families in FL group were *Lachnospiraceae* (26.17%), *Muribaculaceae* (19.48%), *Bacteroidaceae* (17.28%), and *Ruminococcaceae* (9.66%). The main families in FM group were *Muribaculaceae* (22.81%), *Bacteroidaceae* (19.81%), *Lachnospiraceae* (19.04%), and *Prevotellaceae* (12.23%). The main families in FH group were *Lachnospiraceae* (49.70%), *Muribaculaceae* (10.81%), *Bacteroidaceae* (9.38%), and *Ruminococcaceae* (7.01%). Compared with CK group, *Lachnospiraceae*, *Muribaculaceae,* and *Bacteroidaceae* were still the main families in CFBT‐treated samples, and the content of *Lachnospiraceae* increased, while *Muribaculaceae* decreased in FL and FH groups and increased in FM group. This indicated that the CFBT treatment could affect the content of *Lachnospiraceae*, *Muribaculaceae,* and *Bacteroidaceae* in the samples. For different concentrations of CFBT treatment, the main species composition of family level in FM group, in which *Muribaculaceae*, *Lachnospiraceae*, *Prevotellaceae,* and *Bacteroidaceae* were the main families, was the same as that in CK group. The main species composition of family level in FL and FH groups had changed as they were compared with CK group, and the main families in these two samples became *Lachnospiraceae*, *Muribaculaceae*, *Bacteroidaceae,* and *Ruminococcaceae*, indicating that different concentrations of CFBT had different effects on species composition at family level. Compared with the CK group, the main species composition of the NK group changed. The *Lachnospiraceae* content in NK group increased, while the contents of *Muribaculaceae*, *Bacteroidaceae*, *Prevotellaceae,* and *Lactobacillaceae* decreased. Compared with the CK group, the main species composition in YK group was the same. These results indicated that the treatment would affect the change of species composition in samples.

**Table 3:** Distribution of microbiome at family level

Taxon	CK	NK	YK	FL	FM	FH
*Lachnospiraceae*	18.69	40.09	20.96	26.17	19.04	49.70
*Muribaculaceae*	20.80	8.20	23.89	19.48	22.81	10.81
*Bacteroidaceae*	12.11	6.27	18.74	17.28	19.81	9.38
*Prevotellaceae*	17.53	3.73	14.92	7.21	12.23	6.18
*Ruminococcaceae*	10.10	10.70	7.58	9.66	7.23	7.01
*Lactobacillaceae*	12.05	10.94	1.33	6.78	7.12	4.57
*Tannerellaceae*	1.44	5.09	5.85	6.09	4.78	3.28
*Desulfovibrionaceae*	0.45	7.02	0.87	2.07	1.20	2.19
*Helicobacteraceae*	2.62	0.98	1.17	0.37	0.62	0.24
*Burkholderiaceae*	0.16	1.83	0.75	1.09	1.35	0.63
*Rikenellaceae*	1.43	0.72	0.57	0.89	0.65	0.32
*Erysipelotrichaceae*	0.51	0.71	0.78	0.53	0.43	0.94
*Akkermansiaceae*	0.00	0.00	0.78	0.00	0.67	2.43
*Eggerthellaceae*	0.14	1.24	0.53	0.50	0.51	0.31
*Peptococcaceae*	0.34	0.70	0.21	0.36	0.24	0.41
*Family*_XIII	0.15	0.59	0.24	0.36	0.39	0.34
*Saccharimonadaceae*	0.64	0.01	0.23	0.68	0.06	0.08
*Staphylococcaceae*	0.01	0.13	0.05	0.03	0.27	0.72
*Clostridiales_vadinBB60*_group	0.56	0.11	0.06	0.06	0.08	0.01
*Clostridiaceae*_1	0.00	0.16	0.22	0.24	0.11	0.11
*Mitochondria*	0.05	0.35	0.08	0.04	0.09	0.09
*Phaseolus_acutifolius__[tepary_bean]*	0.02	0.27	0.04	0.02	0.15	0.11
*Flavobacteriaceae*	0.14	0.03	0.01	0.01	0.01	0.01
*Enterobacteriaceae*	0.02	0.02	0.04	0.03	0.02	0.04
*Streptococcaceae*	0.04	0.02	0.02	0.00	0.01	0.03
*Bacillaceae*	0.00	0.00	0.01	0.00	0.01	0.00
Unclassified	0.02	0.10	0.07	0.06	0.12	0.04

#### 3.5.3 Changes of Microbiome Structure and Abundance at Genus Level

**Figure 8:**
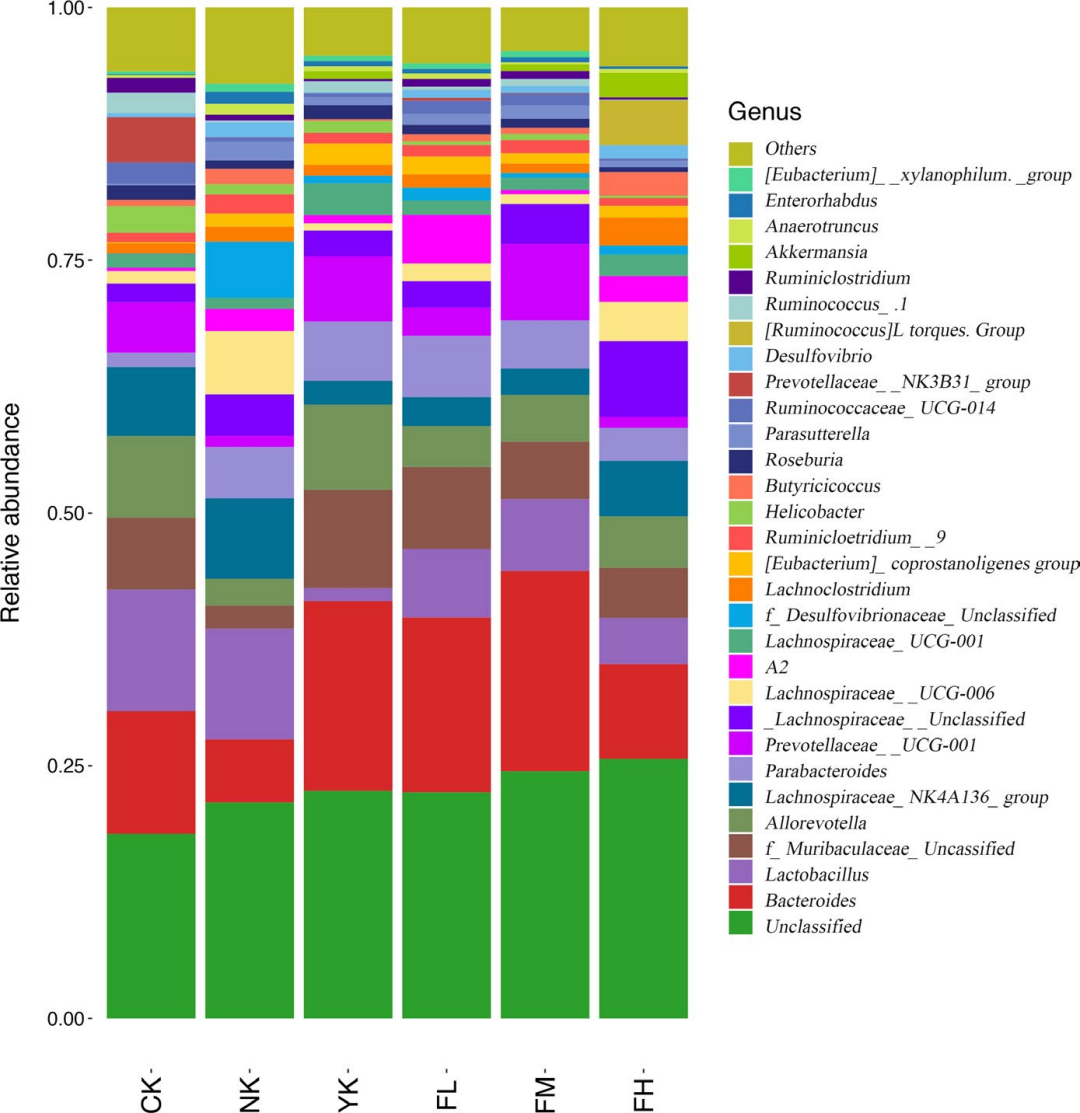
Changes of microbiome structure and abundance at genus level

From the histogram of community structure composition at genus level (Figure 8 and Table 4), the main genera in CK group were *Bacteroides* (12.11%), *Lactobacillus* (12.05%), *Alloprevotella* (8.06%), and *Lachnospiraceae*_NK4A136_group (6.82%). The main genera in NK group were *Lactobacillus* (10.94%), *Lachnospiraceae*_NK4A136_group (7.98%), *Lachnospiraceae*_UCG‐006 (6.29%), and *Bacteroides* (6.27%). The main genera of YK group were *Bacteroides* (18.74%), *Alloprevotella* (8.41%), *Prevotellaceae*_UCG‐001 (6.46%) and *Parabacteroides* (5.85%). The main genera in FL group were *Bacteroides* (17.28%), *Lactobacillus* (6.78%), *Parabacteroides* (6.09%), and A2 (4.77%). The main genera in FM group were *Bacteroides* (19.81%), *Prevotellaceae*_UCG‐001 (7.55%), *Lactobacillus* (7.12%), and *Parabacteroides* (4.78%). The main genera in FH group were *Bacteroides* (9.38%), *Lachnospiraceae*_NK4A136_group (5.47%), *Alloprevotella* (5.10%), and *Lactobacillus* (4.57%). Compared with CK group, although *Bacteroides* and *Lactobacillus* were still the main genera after CFBT treatment, the content of *Lactobacillus* decreased. Besides, *Bacteroides* increased in FL and FM groups but decreased in FH group. This meant that CFBT treatment would affect the content of *Bacteroides* and *Lactobacillus* in the samples. For different concentrations of CFBT treatment, the main species composition in FH group was the same as that in CK group, and *Bacteroides*, *Lachnospiraceae*_NK4A136_group, *Alloprevotella,* and *Lactobacillus* were the main genera in these samples. The main species composition of genus level after CFBT treatment with medium concentration changed as compared with CK group, and the main species of genus level in FM group became *Bacteroides* and *Prevotellaceae*_UCG‐001, *Lactobacillus* and *Parabacteroides*. Compared with CK group, the main species composition of genus level changed after low concentration of CFBT treatment, and the main genera in FL group became *Bacteroides*, *Lactobacillus*, *Parabacteroides,* and A2. These results indicated that different concentrations of CFBT had different influence on species composition at genus level. Compared with CK group, the main species composition at genus level in NK group has changed, and the main species at genus level became *Lactobacillus* and *Lachnospiraceae*_NK4A136_group, *Lachnospiraceae*_UCG‐006 and *Bacteroides*, and the contents of *Bacteroides*, *Lactobacillus* and *Alloprevotella* in NK group decreased, while *Lachnospiraceae*_NK4A136_group and *Lachnospiraceae*_UCG‐006 increased. Compared with CK group, the main species composition at genus level in YK group changed, and the main species at genus level in YK group became *Bacteroides*, *Alloprevotella* and *Prevotellaceae*_UCG‐001 and *Parabacteroides*, and the contents of *Bacteroides*, *Parabacteroides,* and *Prevotellaceae*_UCG‐001 increased while *Lachnospiraceae*_NK4A136_group and *Lactobacillus* decreased, indicating that the treatment could affect the genus composition in these samples.

**Table 4:** Distribution of microbiome at genus level

Taxon	CK	NK	YK	FL	FM	FH
*Bacteroides*	12.11	6.27	18.74	17.28	19.81	9.38
*Lactobacillus*	12.05	10.94	1.33	6.78	7.12	4.57
*f__Muribaculaceae*_Unclassified	7.13	2.29	9.73	8.14	5.66	4.97
*Alloprevotella*	8.06	2.62	8.41	4.04	4.62	5.10
*Lachnospiraceae*_NK4A136_group	6.82	7.98	2.36	2.84	2.62	5.47
*Parabacteroides*	1.44	5.09	5.85	6.09	4.78	3.28
*Prevotellaceae*_UCG−001	4.96	1.06	6.46	2.80	7.55	1.06
*f__Lachnospiraceae*_Unclassified	1.84	4.11	2.54	2.59	3.96	7.52
*Lachnospiraceae*_UCG−006	1.26	6.29	0.73	1.78	0.99	3.86
A2	0.33	2.20	0.79	4.77	0.38	2.56
*Lachnospiraceae*_UCG−001	1.30	1.06	3.17	1.45	1.23	2.13
*f__Desulfovibrionaceae*_Unclassified	0.06	5.58	0.76	1.25	0.48	0.89
*Lachnoclostridium*	1.06	1.48	1.05	1.34	0.91	2.78
*[Eubacterium]_coprostanoligenes*_group	0.11	1.27	2.10	1.76	1.06	1.14
*Ruminiclostridium*_9	0.93	1.94	1.08	1.11	1.26	0.79
*Helicobacter*	2.62	0.98	1.17	0.37	0.62	0.24
*Butyricicoccus*	0.63	1.50	0.17	0.72	0.63	2.34
*Roseburia*	1.41	0.85	1.41	0.92	0.86	0.46
*Parasutterella*	0.16	1.83	0.75	1.09	1.35	0.63
*Ruminococcaceae_*UCG−014	2.12	0.46	0.40	1.31	1.16	0.22
*Prevotellaceae_*NK3B31_group	4.48	0.01	0.01	0.27	0.06	0.02
*Desulfovibrio*	0.39	1.44	0.12	0.82	0.72	1.29
*[Ruminococcus]_torques_*group	0.00	0.00	0.00	0.01	0.01	4.45
*Ruminococcus*_1	2.04	0.20	1.09	0.29	0.65	0.06
*Ruminiclostridium*	1.45	0.59	0.19	0.74	0.80	0.24
*Akkermansia*	0.00	0.00	0.78	0.00	0.67	2.43
*Anaerotruncus*	0.27	1.09	0.49	0.55	0.21	0.39
*Enterorhabdus*	0.10	1.16	0.52	0.46	0.47	0.25
*[Eubacterium]_xylanophilum_*group	0.26	0.83	0.52	0.57	0.63	0.01
Unclassified	18.28	21.35	22.53	22.38	24.46	25.66
Others	6.33	7.51	4.77	5.50	4.30	5.81

#### 3.5.4 Microbial taxa with significant differences between treatment groups

**Figure 9:**
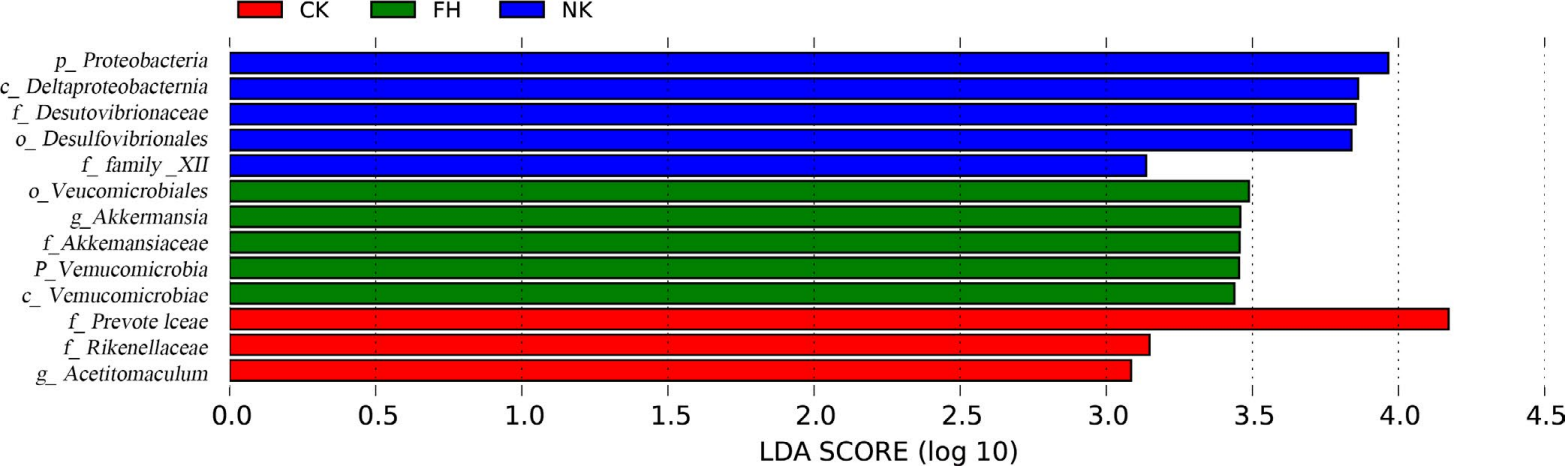
LEfSE diagram of the significant taxonomies between groups. The vertical coordinate is the taxa with significant differences between groups, and the horizontal coordinate is the bar graph to show the LDA difference analysis of each species group. The scores (LDA value is greater than 3) are sorted according to the scores, so as to describe their differences in different groups of samples. The longer the length is, the more significant the difference is. The different colors of the bar chart indicate the sample groups with higher abundance

The difference among fecal microbiome from all groups was analyzed by LDA and the strains with scores greater than 3 were compared. The column distribution of LDA values (Figure 9) shows that only in the three groups (CK, FH, NK) were found the dominant taxa with significant difference, and the numbers of dominant taxa with significant difference were 3, 5, and 5, respectively. The dominant taxa with significant difference in CK were *Prevotellaceae*, *Rikenellaceae*, *Acetitomaculum*; The dominant taxa with significant difference in FH were *Verrucomicrobiales*, *Akkermansia*, *Akkermansiaceae*, *Verrucomicrobia*, *Verrucomicrobiae*; The dominant taxa with significant difference in NK were *Proteobacteria*, *Deltaproteobacteria*, *Desulfovibrionaceae*, *Desulfovibrionales*, Family_XIII.

## 4 DISCUSSION

In human and animal bodies, intestinal microbial balance, intestinal mucosal barrier, and intestinal immune system provide guarantee for the normal barrier function of intestinal tract (Hecht, 1999). Intestinal microbiome provides an important protection against the invasion of foreign microorganisms (Braun & Wei, 2007). The microbial community depends on the competition between different microorganisms and the interdependence and regulation of metabolism to stabilize its composition (Ley, Peterson, & Gordon, 2006). Intestinal microbiome is a large and complex microecosystem, which plays a key role in the homeostasis of the host and directly participates in many functions such as nutrition absorption, energy supply, fat metabolism, immune regulation and disease resistance, and evolves with the host (Backhed, 2005; Kelly, Conway, & Aminov, 2005; Pryde, Duncan, Hold, Stewart, & Flint, 2002).CFBT is a popular tea drink for reducing weight in China. People usually infuse 3 g of dry tea several times with boiling water, which is equal to about dozens of cups of tea. The recommended amount of CFBT for adults is 9 g/day (Zhou et al., 2020). In this study on mice, we used 5, 10, and 20 times of this amount. The HFD‐induced mice were fed with CFBT extraction, and the microbial community and structure in the feces of mice were detected by the Illumina high‐throughput sequencing technology. CFBT administration did not influence food intake (Figure S1), but the diversity and species richness of the samples from different treatment groups were found to be different. In the analysis of intestinal microbiome diversity, CK group had the highest intestinal microbiome diversity, followed by YK group. FL group was also relatively higher, but FM group was lower, and NK group was the lowest, indicating that HFD reduced the diversity of intestinal microbiome in mice, while the positive drug Xuezhikang and CFBT had significant effect on the recovery of intestinal microbiome in mice. However, this recovery effect was not increased with the concentration of CFBT. On the contrary, the recovery effect of low‐dose CFBT was good. In the study of alpha and beta diversity, it was found that CK group had the most abundance and the highest diversity of intestinal microbiome. The microbiome of CK group and CFBT‐treated groups were separated in the PCoA plot. There was a significant difference between these groups, indicating that the CFBT treatment led to the significant change of intestinal microbiome.

In human intestinal tract, there are many kinds of intestinal microorganisms, among which *Firmicutes* and *Bacteroidetes* (Barko, McMichael, Swanson, & Williams, 2018) are dominant. They jointly promote the host to absorb or store energy. The relative abundance of *Firmicutes* has a significant positive correlation with obesity, while *Bacteroidetes* has a significant negative correlation. In this study, compared with NK group, CK and CFBT groups had higher content of *Bacteroidetes* and lower content of *Firmicutes*, which indicated that HFD could significantly reduce the abundance of *Bacteroidetes* and *Bifidobacteria*, and increase the abundance of *Firmicutes*. When the mice were fed with HFD and CFBT, the abundance of *Bacteroidetes* increased significantly and *Firmicutes* decreased significantly. At the family level, *Lachnospiraceae* and *Muribaculaceae* were the dominant bacteria in mice fecal microbiome. Compared with NK group, the content of *Lachnospiraceae* in mice intestinal microbiome decreased in CFBT treatment groups, while the content of *Muribaculaceae*, *Bacteroidaceae,* and *Prevotellaceae* increased, which indicated that the species of intestinal microbiome in mice changed significantly and the abundance of intestinal microbiome increased after CFBT treatment. *Lachnospiraceae* family linked to obesity mainly due to the production of butyric acid, a kind of short‐chain fatty acids (SCFAs) (Kameyama & Itoh, 2014). *Muribaculaceae* may contribute to lipids metabolic disorders and alleviating obesity (Hou et al., 2020). *Prevotellaceae* family produces carbohydrate‐ and protein‐fermenting, acetate, and H_2_, and most strains from this family were enriched in the obese individuals (H. Zhang et al., 2009). However, our result seemed opposite. Maybe the elevation of *Prevotellaceae* needs long‐term consumption of dietary fiber (Méndez‐Salazar, Ortiz‐López, Granados‐Silvestre, Palacios‐González, & Menjivar, 2018). At the genus level, *Bacteroides* and *Lactobacillus* are the main bacteria in the intestinal microbiome of mice. Compared with the NK group, the *Bacteroides* content in CFBT‐treated groups increased, while the *Lactobacillus* content decreased. The contents of *Lachnospiraceae*_NK4A136_group were similar in YK, FH, and FL groups. The content of *Lachnospiraceae*_UCG‐006 was higher than that of NK group. The contents of *Alloprevotella* were similar in CK and YK group, but less in NK group. As saccharolytic bacteria which can produce SCFAs, *Alloprevotella* have been found to improve obesity (Sharma, Li, Stoll, & Tollefsbol, 2020). The content of *Parabacteroides* was higher in the low‐dose group (FL). Bile acids were negatively correlated with *Parabacteroides* which were elevated after HFD feeding (Lin, An, Hao, Wang, & Tang, 2016). It has also been found that *Bifidobacterium*, *Lactobacillus,* and *Bacteroidaceae* can produce bile acid hydrolase (BSH), which can transform conjugated bile acid into free bile acid, affect the enterohepatic circulation of bile acid, promote the synthesis of bile acid by the liver using cholesterol, make more cholesterol in the blood be converted, and realize the effect of lowering blood cholesterol (Bhange, Sridevi, Bhange, Prabhune, & Ramaswamy, 2014; Junguo et al., 2013; Li, 2012; Miremadi, Ayyash, Sherkat, & Stojanovska, 2014). The dominant taxa with significant difference in FH group, *Akkermansia* (phylum *Verrucomicrobia*), have a negative correlation with obesity through degrading intestinal mucin (Depommier et al., 2019). Among the dominant taxa with significant difference in NK group, the family *Desulfovibrionaceae* (*Proteobacteria* phyla) was positively associated with obesity (Delzenne & Cani, 2011), and Family_XIII strains have been implicated to produce butyrate, a SCFA, and positively correlated with 3‐OH‐butyrate levels in obese individuals (Schmidtner et al., 2019). Therefore, CFBT, to a certain extent, can play a role in reducing hyperlipidemia. In addition, the effect of tea on improving the structure of intestinal microbiome has been widely recognized. Tea also has obvious inhibitory effect on common pathogenic bacteria and harmful intestinal bacteria and can promote the growth of intestinal probiotic *Lactobacillus* (Xianglan, 2013). The active ingredients in tea have a regulatory effect on the intestinal microbiome. Some studies have shown that the level of *Lactobacillus* in fecal microbiome has been significantly improved after tea polyphenol was fed to animals (Okubo et al., 1992). It has been found that tea polyphenol can selectively inhibit some bacilli in large intestine, maintain the balance of intestinal microbiome, and is beneficial to intestinal health.

In conclusion, CFBT can increase the abundance of intestinal microbiome in mice, regulate the disorder of intestinal microbiome caused by high‐fat diet, promote the growth of beneficial bacteria to a certain extent, reduce the number of pathogenic bacteria, and keep the host health.

## Conflict of Interest

The authors declare that they do not have any conflict of interest.

## ETHICAL STATEMENT

Ethical Review: This study was approved by the Animal Experiment Ethics Committee of Hunan Agricultural University. The ethical approval code is SCXK (Xiang) 2016–0002.

## Supporting information

Fig S1
