# Supplementary figures and images for: Compound Fu brick tea modifies the intestinal microbiome composition in high‐fat diet‐induced obesity mice
Source: Food Sci Nutr. 2020 Aug 31;8(10):5508–20. doi: 10.1002/fsn3.1850 (PMC7590332; doi:10.1002/fsn3.1850)

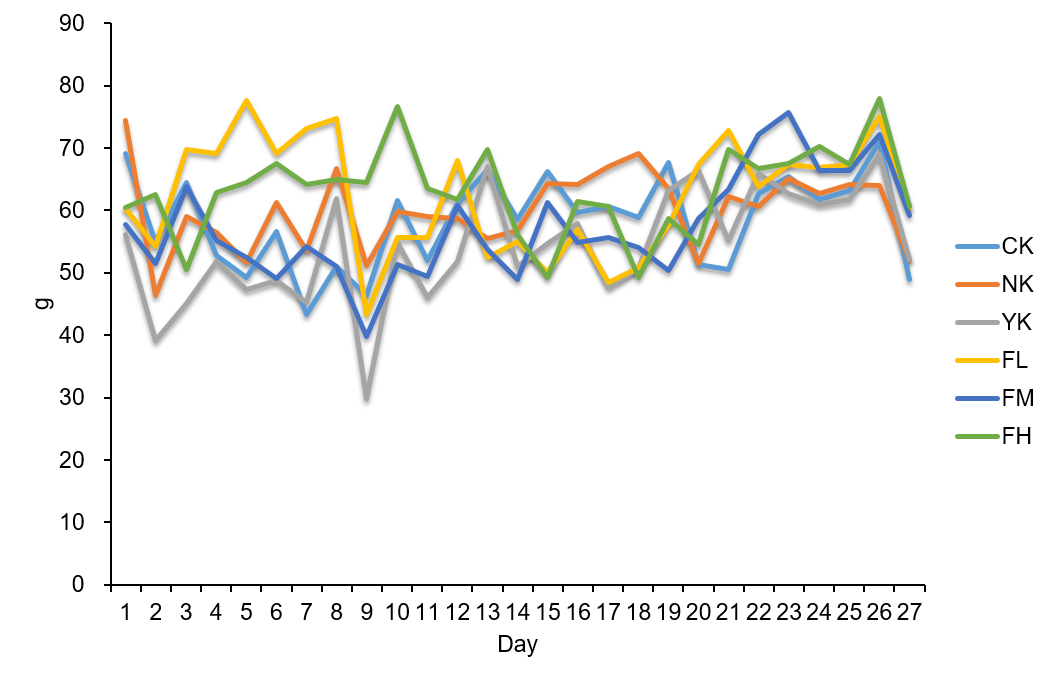

Supplement: Supplementary file 1 — Fig S1 [file FSN3-8-5508-s001.tif]
